# Vikram Patel: fresh ways of delivering mental health care

**DOI:** 10.2471/BLT.23.030523

**Published:** 2023-05-01

## Abstract

Vikram Patel talks to Gary Humphreys about developing evidence-based approaches to collaborative mental health service provision.

*Q: Since we last interviewed you (2017), Sangath has focused significant resources on meeting the demand for mental health services for India’s adolescents. Can you tell us something about that work?*

*A*: It grew out of a project we initiated with children back in the late 1990s. As you may recall, Sangath started out with a child guidance clinic in Goa, where we quite quickly became aware of the limitations of our delivery model. A huge number of families came to the clinic to get an assessment of their child, but we also observed very high dropout rates. There were a number of reasons for this, among them parents being reluctant to commit to extended treatment schedules. We also came to realize that, despite the lines of people that were often outside the doors of the state’s only psychiatric institution, many families were still staying away because of the stigma associated with mental illness and the fear inspired by decades of carceral, asylum-centred tradition.

We knew that in order to meet the mental health needs of the population, we had to find some way to engage with people in their own homes. We began to look at different approaches to delivering care, and hit on one which had been used with great success in maternal and child health. This involved empowering ordinary people and community health workers to deliver mental health care, with training and supervision from experts. So, we were early adopters of an approach that is now widely used and backed by a body of evidence that includes randomized controlled trials done in more than 40 countries, demonstrating the effectiveness of task-sharing for prevention and care of a range of mental health conditions. In our case, we were able not just to demonstrate how the provision of mental health care could be scaled, but also how it could be delivered to adolescents, a group that was, and still is, widely neglected despite being the most at-risk for the development of mental health problems. It is also worth remembering that India is home to an estimated 250 million adolescents, around 20% of the total global population of 10–19-year-olds.

*Q: Adolescents are different to children in many ways. How did you adapt your approach to them?*

A: The key strategies were to meet young people on their own ground, in particular in their schools, and to design interventions that could be implemented by front-line providers. They also had to be developmentally appropriate – which is to say, reflecting the differences you allude to, including for example the desire for greater autonomy. We used funding and other support from a principal research fellowship that I had been awarded by the Wellcome Trust to develop a suite of transdiagnostic psychological interventions (suitable for a variety of mental health presentations) that were designed to be delivered in three to four sessions over three weeks in school settings.

“Why […] are we not seeing better [mental health care] results at the population level?”

*Q: Can you describe the intervention?*

A: The core intervention was based on a developmentally informed, problem-solving technique that is an integral component of cognitive behavioural therapy (a therapy designed to change cognitive distortions and associated behaviours, and help develop coping strategies). We broke the process down into three simple-to-understand steps, comprising identification of the problem, optional responses, and action – what we referred to as the ‘Problems-Options-Do it’ exercise. Essentially, the exercise was designed to help adolescents identify the problems that were causing stress, and work on those problems rather than becoming mired in distressing emotions or acting impulsively in negative or harmful behaviours. The intervention was delivered by a school-based lay counsellor and supported with self-help materials in the form of printed booklets which explained problem-solving using illustrated vignettes and also described corresponding home practice exercises. We tested the approach in schools in low-income neighbourhoods in New Delhi between 2017 and 2020.

*Q: How effective was it?*

A: A clinical trial with a 12-month follow-up indicated that it helped reduce self-reported psychosocial problem severity in the subjects, and these effects were sustained for at least a year.

*Q: What happened to participants who continued to experience problems?*

A: They were offered a more tailored, modular, higher-intensity second step, involving personalized psychological treatment. Unfortunately, our work on that second step was disrupted by school closures during the pandemic and more research is needed to evaluate it. Our feeling is that a stepped care delivery model of mental health care in which the least resource-intensive treatment is offered to all those in need of it, while more resource-intensive and specialist care is reserved for those who do not respond, has the potential to be both scaleable and cost-effective.

*Q: You reference school closures during the pandemic. What impact have they had on the mental health of children and adolescents in India?*

*A: *There are now dozens of studies which have reported a significant increase in the incidence of anxiety and depression, particularly in young people. It appears that we are slowly returning to pre-pandemic levels, but I fear that the cohort of young people who were at a critical developmental stage of their life in the early years of the pandemic which were marked by lockdowns and school closures, will experience impairments or challenges that may have lifelong implications.

Schools and educational institutions are not simply places where young people go to study, they are spaces in which they socialize with their peers, possibly escape difficult home circumstances and often receive their main meal of the day. Given all that, and knowing what we did about the importance of in-person peer contact for shaping the mental health of young people, we really need to ask ourselves if we could not have tried harder to find an alternative to the total shutting down of their lives and education. For example, could schools have been excluded from lockdowns or could they have been run outdoors? This is a question that I think policy-makers need to address in preparation for the next respiratory pandemic. The pandemic has of course also presented some silver linings, notably by bringing mental health issues into the public spotlight, and by accelerating the acceptance and application of digital tools in health care.

*Q: To what extent has Sangath made use of digital technologies in engaging with adolescents?*

A: We developed a blended problem-solving game-based intervention for adolescents which is designed to support the learning of problem-solving techniques in a fun, self-guided way, possibly with facilitation by a school counsellor or teacher. We are also making use of social media to engage the youth community in India on mental health-related topics, including storytelling as a way of challenging the myths and stigma attached to mental illness.

It’s perhaps worth noting that Sangath was in fact an early adopter of digital technologies and applies them in diverse ways. For example, with regard to the school-based problem-solving intervention just described, we have used e-learning platforms to help front-line providers learn how to deliver this treatment. We are also taking this approach in a new initiative which aims to rapidly expand the workforce capable of delivering evidence-based psychosocial interventions.

“Schools and educational institutions are not simply places where young people go to study.”

*Q: Can you tell us more about it?*

A: It uses digital tools that enable frontline providers to learn and master evidence-based psychosocial interventions and deliver them as a part of quality-assured care. We are using a two-step structure, the first step focusing on didactic content with specific modules for various topics and lessons, including objectives for each lesson and source material. The second step focuses on mastering the skills needed to deliver the intervention through a case-based internship.

*Q: Have you had a chance to test it in the field?*

*A: *To date most of the foundational work has been done in India, led by Sangath in partnership with local and international institutions. For example, in the central state of Madhya Pradesh, one of the least resourced states in India, the intervention is being implemented in partnership with the state government’s health department, and involves the training of about 500 accredited social health activists (ASHAs), all of whom are women and who serve as the frontline providers of the country’s National Health Mission. ASHAs were originally intended to help improve maternal and child health outcomes in rural communities, but their role has now expanded to addressing issues related to noncommunicable diseases and, most recently, to the door-to-door COVID-19 vaccination campaign. And now they are working with us on mental health.

*Q: Are there any plans to test the intervention in other countries?*

A: Absolutely, and we are not limiting ourselves to low- and middle-income countries, since resource constraints can occur across the country income spectrum. So, for example, building on the lessons learned in India, we are now working in Texas in the USA, where there are also gaps in access to, and availability of, quality-assured mental health services. Working in partnership with the Meadows Mental Health Policy Institute and the University of Texas Southwestern Medical Center, and with money from the Lone Star Prize, we are implementing the intervention to scale up depression care in under-resourced communities. Over the next three years, we are hoping to recruit, train and support hundreds of frontline providers in the delivery of behavioural activation, an evidence-based psychotherapy for depression. My hope is that work we are doing in Madhya Pradesh and Texas will generate real-world case studies replete with learning opportunities, to inform and refine future implementation strategies. The project is the culmination of nearly two decades of work I did in India, which I now hope can be of benefit in diverse global contexts where there is also a massive unmet need for mental health care and where people are asking themselves, “Why, with all the money we spend on mental health specialists, are we not seeing better results at the population level? Do we need a fresh way of thinking about mental health care?”

